# Regional simulation of soil organic carbon dynamics for dry farmland in Northeast China using the CENTURY model

**DOI:** 10.1371/journal.pone.0245040

**Published:** 2021-01-19

**Authors:** Feng Zhang, Shihang Wang, Mingsong Zhao, Falv Qin, Xiaoyu Liu

**Affiliations:** 1 School of Surveying and Mapping, Anhui University of Science and Technology, Huainan, China; 2 School of Geography and Land Engineering, Yuxi Normal University, Yuxi, China; 3 School of Information Engineering, Jiangsu Vocational College of Agriculture and Forestry, Jurong, China; Universidade de Santiago de Compostela, SPAIN

## Abstract

Soil organic carbon content has a significant impact on soil fertility and grain yield, making it an important factor affecting agricultural production and food security. Dry farmland, the main type of cropland in China, has a lower soil organic carbon content than that of paddy soil, and it may have a significant carbon sequestration potential. Therefore, in this study we applied the CENTURY model to explore the temporal and spatial changes of soil organic carbon (SOC) in Jilin Province from 1985 to 2015. Dry farmland soil polygons were extracted from soil and land use layers (at the 1:1,000,000 scale). Spatial overlay analysis was also used to extract 1282 soil polygons from dry farmland. Modelled results for SOC dynamics in the dry farmland, in conjunction with those from the Yushu field-validation site, indicated a good level of performance. From 1985 to 2015, soil organic carbon density (SOCD) of dry farmland decreased from 34.36 Mg C ha^−1^ to 33.50 Mg C ha^−1^ in general, having a rate of deterioration of 0.03 Mg C ha^−1^ per year. Also, SOC loss was 4.89 Tg from dry farmland soils in the province, with a deterioration rate of 0.16 Tg C per year. 35.96% of the dry farmland its SOCD increased but 64.04% of the area released carbon. Moreover, SOC dynamics recorded significant differences between different soil groups. The method of coupling the CENTURY model with a detailed soil database can simulate temporal and spatial variations of SOC at a regional scale, and it can be used as a precise simulation method for dry farmland SOC dynamics.

## Introduction

Soil fertility and grain yield are significantly affected by soil organic carbon content (SOCC), thus making it important for agricultural production and food security [1]. The soil carbon pool is the second largest global carbon pool after the oceans, accounting for 66.7% of the total terrestrial ecosystem organic carbon pool [2]; The potency of carbon dioxide in the air was easily affected by SOCC in farmland soil [3]. Previous investigations have shown that surface soil organic carbon in China accounts for 4.4% of the global surface soil total (19.6 Pg) while the total global organic carbon pool is estimated at 49 Pg [4]. Under natural conditions, the regulation of soil on carbon dioxide is slow, while dryland soil can regulate the SOCC in a relatively short time due to the strong influence of human activities.

As changes in soil organic carbon (SOC) is a complex process, models are accepted as the most suitable way to estimate the evolution of organic carbon in soil. Currently, the RothC model, the DNDC model and the CENTURY model have been widely used. The CENTURY model has been applied to different terrestrial ecosystems, such as timberland, farmland and grassland ecosystems [5]. Moreover, this model has been applied and verified using measured data obtained from long-term soil monitoring points in different countries [6–10]. In addition, the application of the model at regional scale or national scale were successful [11–15]. In China, SOC dynamics for cropland were initially simulated using the CENTURY model in the 1980s, and numerous studies have used this model to reliably simulate SOC dynamic changes on the point scale [16–20]. In China, dry farmland is the main farmland land use type, and its SOCC is relatively low [21].

Due to the vast area of dry farmland soil and low SOCD, its carbon sequestration potential is significant. As dry farmland may have a significant influence on global climate change, it is therefore important to accurately estimate its SOC dynamics on a regional scale. Currently, only a few simulation studies on the dynamic changes of SOCD and reserves in the dry farmland of Jilin Province have been undertaken; studies examining potential of soil carbon sequestration have only been undertaken by Wang et al [22] and Yu et al [23]. The contents of our study, therefore, were to:

1. Dynamic simulation and verification of SOC in dry farmland at point scale.
2. Simulate regional scale temporal and spatial SOC dynamics of dry farmland in Jilin Province from 1985 to 2015 using the CENTURY model.
3. Identify differences in the evolution of SOC between different soil types in Jilin Province.

## Materials and methods

### Study area

**Fig 1:**
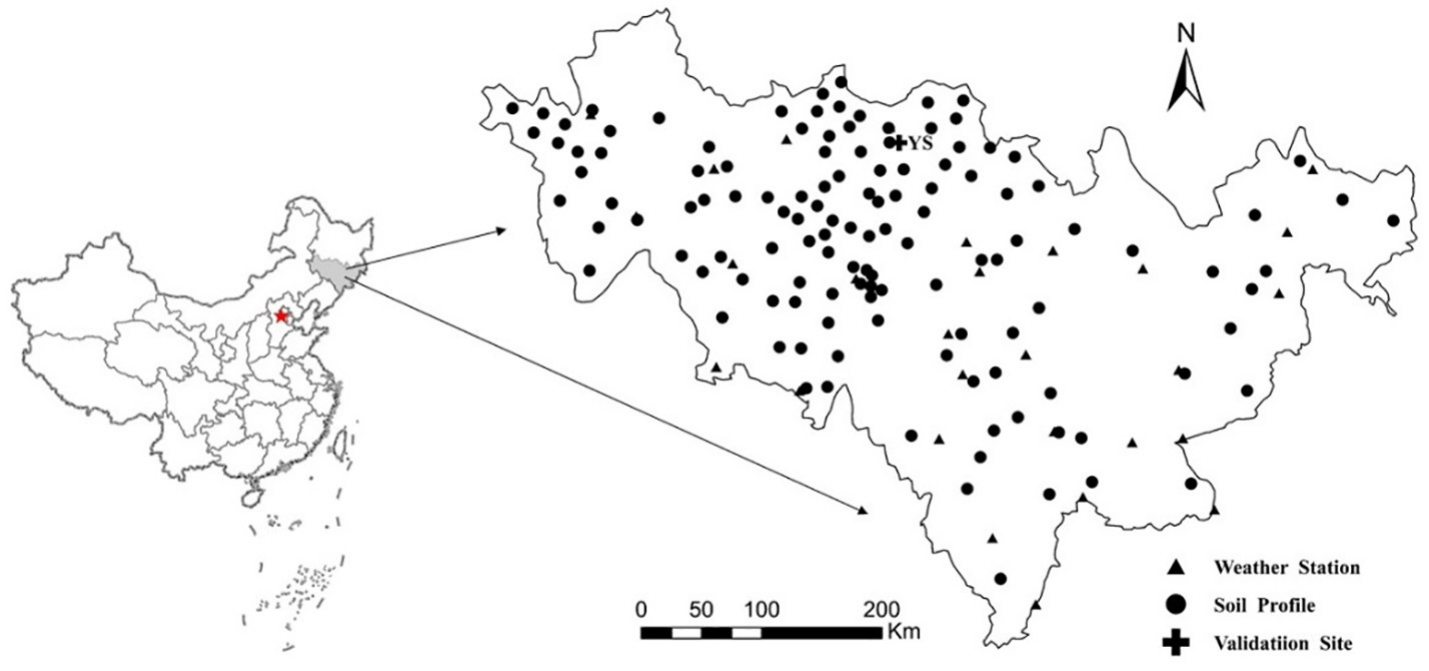
Geographical location of the study area indicating soil profile points and weather stations in Jilin Province, China.

Jilin Province (40°50’-46°19’ N, 121°38’-131°19’ E), located in the central part of northeast China (Fig 1), has rich mineral and agricultural resources. Terrain in this province inclines from the southeast to the northwest, having characteristics of high levels in the southeast and low levels in the northwest. The province spans five major rivers: Tumen River, Yalu River, Liaohe River, Suifen River and Songhua River. Jilin Province has a temperate continental monsoon climate, having distinct seasons. Mean annual temperature ranges from 2°C to 6°C and the frost-free period is generally 100–160 days.

Although the region has 2259–3016 average sunshine hours and an annual average precipitation of 400–600 mm, seasonal and regional differences are large. The total area of soil resources in Jilin Province is 186,500 km^2^. Soil types in the province include 19 types and 263 soil species. The capital soil groups for dry farmland in the province, as represented in the Genetic Soil Classification of China (GSCC) [24], are dark brown soil, black calcareous soil, white pulp soil, meadow soil, black soil, aeolian sandy soil, newly accumulated soil and saline-alkali soil, referencing to the FAO/UNESCO taxonomy [25]. The total area of these eight soil types accounts for 93.79% of the total area of soil resources in the province; the remaining 11 soil types account for only 6.21%. The cultivated soil in this province is mainly composed of black calcareous soil, meadow soil, white pulp soil, black soil and dark brown soil. The cultivated land area of Jilin Province is about 6.99 million ha, of which dry land accounts for about 80%. Corn and soybean are the main crops planted, and the ripening system is once a year. The main fertilizers are chemical fertilizer and organic fertilizer.

### Model description

The CENTURY model was established by Parton et al [26] in 1983 to simulate long-term dynamic changes of carbon, nitrogen, phosphorus and sulfur in a grassland ecosystem. After continuous improvement, the model was gradually applied to other terrestrial ecosystems, such as farmland and forest. The CENTURY model is a biogeochemical cycle model which integrates several sub-models, such as the soil organic matter (SOM) sub-model, the plant productivity sub-model, soil nitrogen, phosphorus and sulfur cycle sub-models, and soil temperature and water molecular models. This model takes into account the effects of soil, climate and human management measures on plant productivity and biomass. The main parameters of the operation model include soil parameters (soil texture, thickness, bulk density, pH etc.), climate parameters (monthly mean maximum and minimum temperature, monthly precipitation, etc.) and crop management parameters (crop species, tillage mode, chemical fertilizer species and quantity, harvesting mode, organic fertilizer species quantity, planting time, crop start growth time, crop end growth time, etc.). When this model is used to simulate different ecosystems, it is important to localize the model parameters. For a detailed introduction about CENTURY model refer to Parton et al. [26].

### Polygon-based database

By using ArcGIS 10.2 (ESRI, 2013), spatial overlay analysis was used to create a soil attribute database (with 1282 polygons) incorporating soil data from soil and land layers (at a 1:1,000,000 scale). In addition, a corresponding soil attribute database was established with reference to soil species in Jilin. This database contained attribute data of 126 soil profiles, these being the most typical soils in the different distincts, and they were classified according to the GSCC [22]. Sampling time for the soil profile data was taken as the second Soil Census in China. By adopting the pedological knowledge based (PKB) method proposed by Zhao et al [27], a 1:1,000,000 soil database containing soil attribute data was established. Finally, the soil polygons representing the soil profiles (Fig 1) were created to support the CENTURY simulations in the province.

### Meteorological data

Twenty eight meteorological stations (Fig 1), provided by the National Meteorological Information Center, China Meteorological Administration (NMIC-CMA). Data collected by these stations included monthly precipitation, annual sunshine hours, and monthly average maximum and minimum temperatures. According to the principle of nearest neighborhood, the meteorological data for each polygon was determined by the nearest meteorological station.

### Model parameterization and initialization

The CENTURY model divides the total soil organic matter (SOM) pool into three types: active carbon pool, slow carbon pool and inert carbon pool. Although the proportion of the active carbon pool constitutes the greatest amount, the volume of this type differs in each carbon pool. Therefore, the decomposition rate of the three carbon pools significantly differs from the turnover time of organic carbon. As the CENTURY model is highly sensitive to the distribution of initial SOM in the three carbon pools, therefore the parameters of the model must be initialized before the model is run at different scales. According to the initialization method of Ogle et al. [28], parameter initialization of the model was divided into three stages. (i) First stage: assuming the landscape is in a natural state, and not affected by human activities, the model was run for 3000 years (4000 BC-1000 BC) to accumulate SOC from 0 to a relative equilibrium state. The original vegetation was assumed to be C3 grassland in a temperate zone, with moderate grazing and 1 fire in 10 years; meteorological data used was the average value of meteorological data of 26 years from 1955 to 1980 collected from stations near the monitoring point. Soil data, such as soil texture in the 0–20 cm layer, pH and soil bulk density, were derived from monitoring data. (ii) Second stage: from 1000 BC to 1960, human activities resulted in changes in land use patterns and agricultural production patterns. Therefore, the model was run to simulate the effects of land use patterns and human production activities on SOC. During this period, only a small amount of organic fertilizer was applied to dry farmland soil. (iii) Third stage: from 1960 to 1985, a small amount of chemical fertilizer and organic fertilizer were used in agricultural production at the same time. By adjusting the parameters of each stage, such as potential crop productivity (PRDX (1)), the simulated value of SOC at the end of initialization was close to the measured value of SOC at the beginning of our monitoring period.

### Calculation of SOC

SOC at each monitoring site was described as SOCC (g kg^−1^). Here, each polygon (0–20 cm) participated in the calculation and SOCC was changed into SOCD, (Mg C ha^−1^), according to the following formula: [9, 15]

$$SOCD_{i}=SOCC_{i\;}*BD_{i}*H*0.1$$ (1)

where, *SOCD*_*i*_ is the soil organic carbon density (Mg C ha^−1^) of the polygon; *BD*_*i*_ is the bulk density (g cm^-3^) of the polygon; and *H* is the layer thickness. In our study, *H* was set to 0–20 cm.

Soil organic carbon stocks (SOCS, Tg = 10^12^ g) were count as:

$$SOCS=\sum_{i=1}^{1282}\frac{SOCD_{i}*S_{i}}{10^{6}}$$ (2)

where, *SOCD*_*i*_ and *S*_*i*_ are the SOCD and the area (ha) of each polygon, respectively.

Difference value of SOCD from 1985 to 2015 was calculated as:

$$\Delta \text{SOCD}=\text{SOC}{\text{D}}_{2015}-\text{SOC}{\text{D}}_{1985}$$ (3)

where, *SOCD*_*2015*_ (Mg C ha^−1^) and *SOCD*_*1985*_ (Mg C ha^−1^) are the soil organic carbon density, which were simulated values obtained using the CENTURY model for each polygon in the year 2015 and 1985.

### Monitoring point data

Site information provided by the National Agricultural Technology Extension and Service Center of China included soil texture (sand, silt and clay), soil organic matter (SOM), bulk density, pH, crops rotation and management practices (Table 1). The site scale simulation can provide reference for further calibration of the model parameters. In addition, it could reflect the change trend of SOC in the region to a certain extent.

**Table 1:** Site characteristics (0–20 cm) for the field-validation site in Jilin Province.

name	Location	Year	Crop	pH	Bulk density	Texture			SOCD	Soil
						Sand	Silt	Clay		
					g cm^-3^	-------%-------			g m^-2^	
Yushu	126°3’00" E	1988–2007	corn	5.0	1.00	45.0	34.5	18.7	2413	black soil
	44°49’48" N									

Validation of simulation of the CENTURY model using data from a long-term monitoring site (YS) was performed by analyzing the correlation between simulated values and measured values. To assess whether simulated values follow the same pattern as measured values, the sample correlation coefficient (*r*) of linear regression analysis was calculated as:

r=∑i=1n(Oi−O¯)(Pi−P¯)[∑i=1n(Oi−O¯)2]12[∑i=1n(Pi−P¯)2]12 (4)

where, *P*_*i*_ is the simulated value; *O*_*i*_ is the measured value; *Ō* is the average of measured values; and n is the number of data pairs. A high correlation was shown by *r* ≥ 0.8; values between 0.5 and 0.8 indicated moderate correlation; values between 0.3 and 0.5 indicated a low or weak correlation; and values < 0.3 indicated an extremely weak correlation, or no correlation.

Apart from the correlation coefficient, an index modeling efficiency (ME) [6] was calculated as:

$$ME=1-\frac{\sum_{i=1}^{n}{(P_{i\;}-O_{i})}^{2}}{{\sum_{i=1}^{n}\left(P_{i}-\bar{O}\right)}^{2}}$$ (5)

where, *ME* values equal to 1 indicated a perfect fit and values below 0 indicated a closer fit.

## Results and discussion

### Model validation results

**Fig 2:**
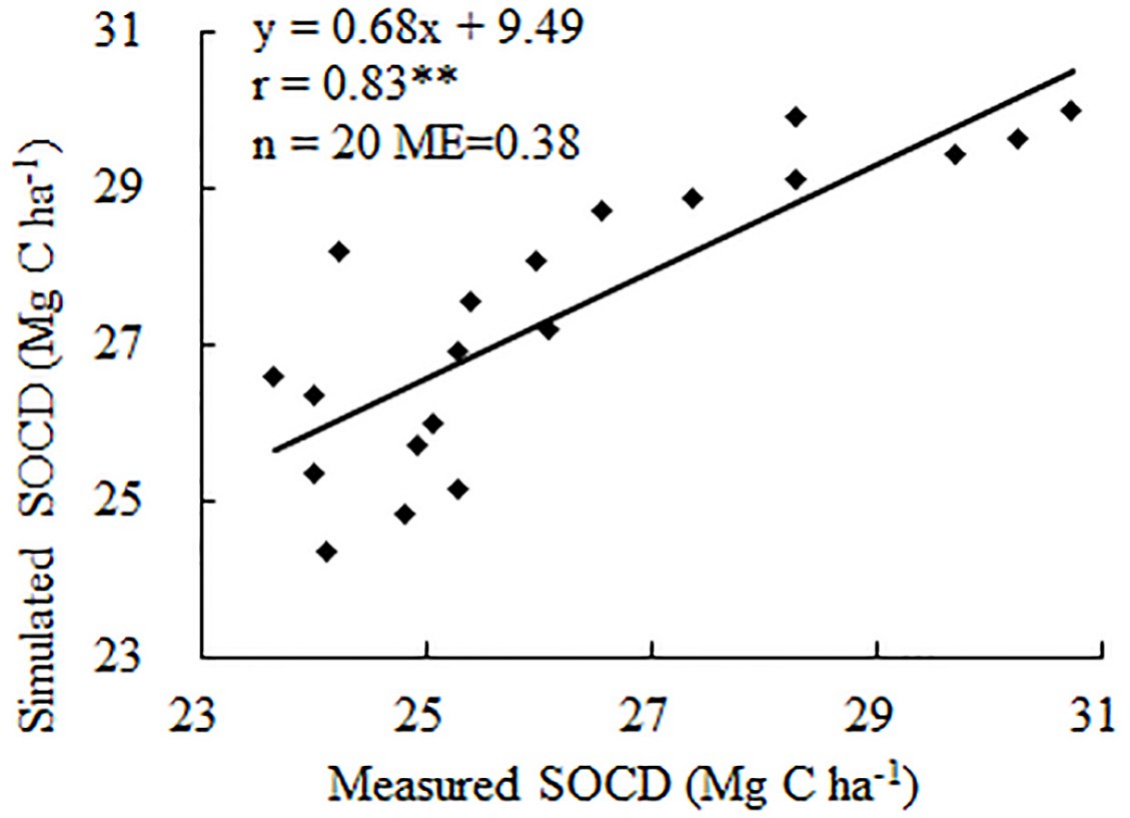
The relationship between measured and simulated SOCD at Yushu site, Jilin Province, China. ** Significant P<0.01level. ME = modeling efficiency.

Correlation analysis at the long-term monitoring site (YS) indicated a significant linear relationship (*P* < 0.01, *r* = 0.83 and *ME* = 0.38) in Fig 2. Our results therefore showed that the model accurately simulated SOCD at the long-term monitoring site. In addition, SOC dynamics at the site were satisfactorily represented by simulation using the CENTURY model.

### Simulation statistics of soil polygons

SOCD descriptive statistics for all soil polygons in 1985 and 2015 are shown in Table 2. It can be seen that minimum SOCD increased from 1985 to 2015, and maximum SOCD decreased over the same time. The range of SOCD value was 194.8 Mg C ha^−1^ in 1985 and 153.11 Mg C ha^−1^ in 2015. Compared with SOCD in 1985, the mean value in 2015 decreased by 1.65 Mg C ha^−1^, indicating that SOCD of dry farmland tended to be more concentrated over the past 30 years.

**Table 2:** SOCD descriptive statistics for 1985 and 2015.

Item	Numbers	Minimum	Maximum	Mean	Range	Standard deviation	Variance
		-----------Mg C ha^−1^-----------					
SOCD1985	1 282	8.00	202.80	39.54	194.80	23.83	567.86
SOCD2015	1 282	12.29	165.40	37.89	153.11	20.52	421.07

**Fig 3:**
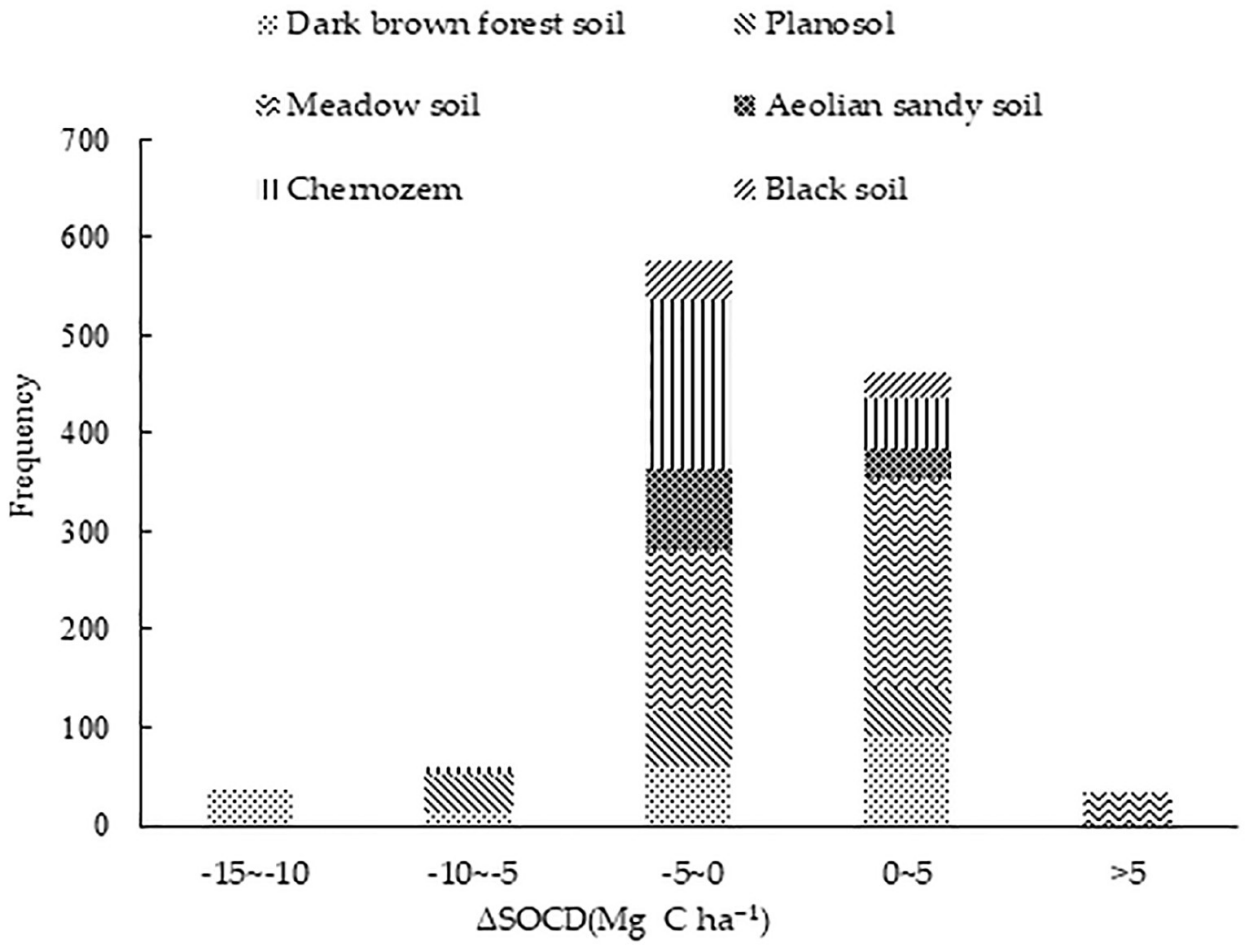
Frequency distribution of ΔSOCD in different soil groups in Jilin Province, China.

The difference between initial and final SOCD simulated value is shown as ΔSOCD. The simulation results show that (Fig 3), the ΔSOCD in Jilin Province had a large range (43.59 Mg C ha^−1^). This shows that the SOCD in Jilin Province differed greatly, and the range of change was not stable. The minimum and maximum ΔSOCD was -37.40 Mg C ha^−1^ (peat soil) and 6.19 Mg C ha^−1^ (brown soil), respectively. The soil polygons with a negative ΔSOCD (indicating a loss of carbon) accounted for 60.04% of all polygons. Consequently, soil fertility decreased. In this study, 549 soil polygons recorded positive ΔSOCD values, with 42.50% of these positive sites being located on meadow soil. According to statistical results of the polygons, SOC in Jilin Province showed a downward trend during the simulation period.

628 polygons with a ΔSOCD value between -5 to 0 Mg C ha^−1^ had an area covering 3.4×10^6^ ha, accounting for 60.13% of all soil polygons. 473 polygons with a ΔSOCD value ranging between 0 and 5 Mg C ha^−1^ were located in an area spanning 1.8×10^6^ ha, accounting for 32.56% of all soil polygons. Only 3.4% of soil polygons showed an increase of SOCD of more than 5 Mg C ha^−1^. The SOCD in the same soil type recorded both increases and decreases, with the extend of change not being the same. The decrease of SOC was related to the area of soil type. A large proportion of negative ΔSOCD values could be attributed to carbon loss from polygons in the chernozem area (41.61%). If SOCD in Jilin Province is in a long-term decline, dry farmland production will decline, not being conducive to sustainable soil use.

### Temporal dynamics of SOC

**Fig 4:**
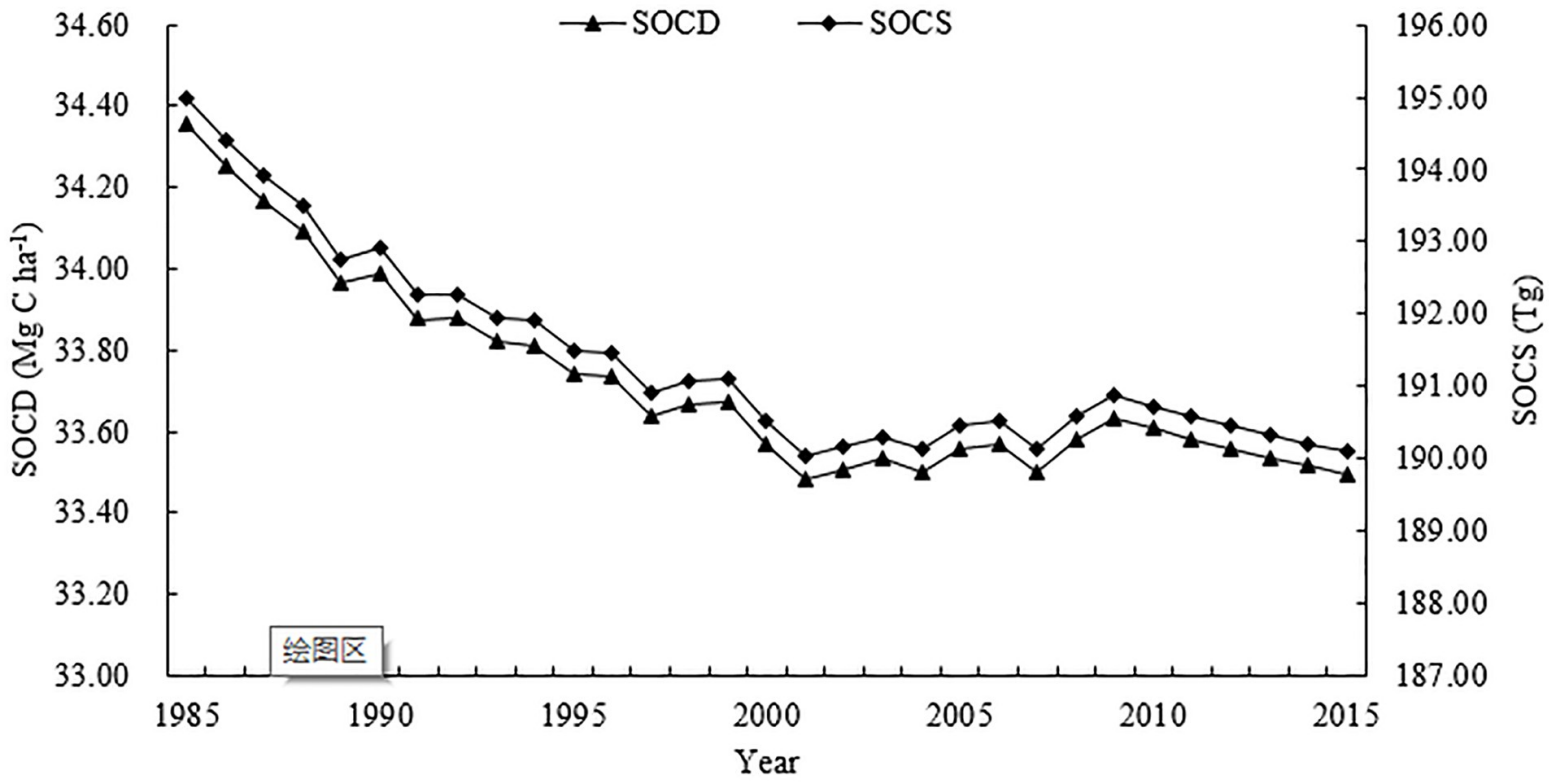
The temporal change of soil organic carbon density (SOCD) and soil organic carbon stock (SOCS) in dry farmland in Jilin Province, China.

The temporal changes of SOC indicated that SOCD in Jilin Province decreased from 34.36 Mg C ha^−1^ in 1985 to 33.50 Mg C ha^−1^ in 2015 (Fig 4), a decrease of 0.86 Mg C ha^−1^ with an average rate 0.03 Mg C ha^−1^ per year. Over the same time period, SOCS decreased from 194.99 Tg to 190.02 Tg. Results for simulated SOCS were similar to the result of 206 Tg for cultivated land calculated by Yu et al [23]. The result of Yu et al. was greater than the simulated result due to paddy soils in the research area which had an approximate SOCS value of 16.54 Tg within the 0–20 cm depth. Although the density and stocks of SOC increased in some years, SOCD and SOCS have continued to decline over the past 30 years. Therefore, in the future, SOCD in dry farmland may have a larger growth space under the scientific farmland management measures.

SOCS was 194.99 Tg in 1985 while 190.10 Tg in 2015 (Fig 4), having a decrease of 0.16 Tg C per year. A decrease of 4.89 Tg SOC, amount to 17.71 Tg of CO_2_, has been released into the atmosphere. This indicates that dry farmland in Jilin Province has been a carbon source during simulation period. Although the trend of carbon deterioration in dry farmland soils in Jilin Province has previously been identified [29, 30], some research methods and area of investigation were not completely the same as those in our study. As our study is based on detailed soil data from the fine-scale 1:1,000,000 soil database, our results should therefore improve previous estimates.

### Spatial distribution of SOC

**Fig 5:**
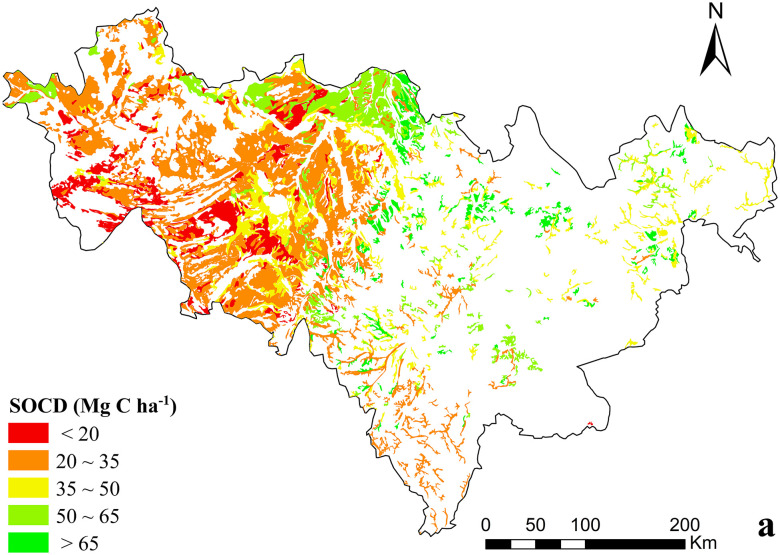
Spatial variability of SOCD of dry farmland in1985 of Jilin Province, China.

Dry farmland of Jilin Province was mainly distributed in the central and western parts (Fig 5). In 1985, SOCD in Jilin Province was higher in the east and lower in the west, and higher in the north and lower in the south. More than half of farmlands (51.01%), its SOCD was between 20–35 Mg C ha^−1^. SOCD of dry farmland ranging from 35 to 50 Mg C ha^−1^ covered 16.90%. The proportion of dry farmland with an SOCD value between 50 and 65 Mg C ha^−1^ was 13.14%. About 12.83% of the total area had an SOCD value less than 20 Mg C ha^−1^, and these soils were mostly distributed in the western area of Jilin Province. Only 6.13% of soils had an SOCD bigger than 65 Mg C ha^−1^, which were scattered throughout the north of the province and the eastern area.

**Fig 6:**
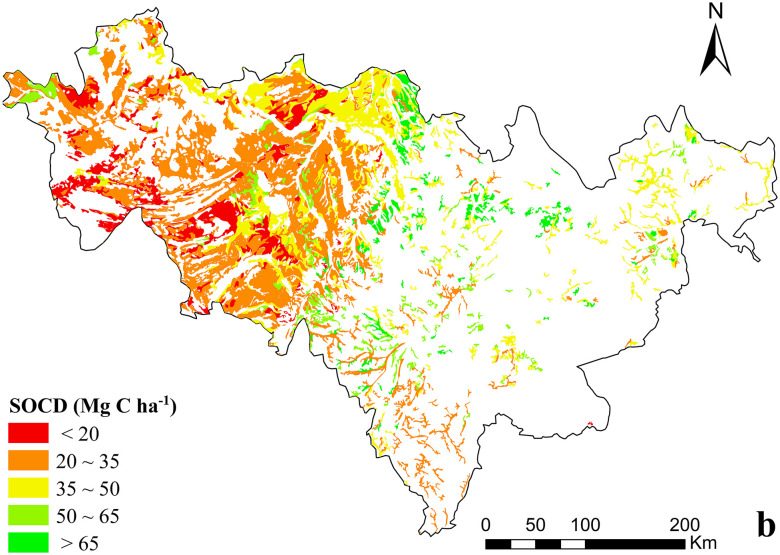
Spatial variability of SOCD of dry farmland in 2015, Jilin Province, China.

In 2015, the proportion of dry farmland with SOCD between 20–35 Mg C ha^−1^ (Fig 6) was51.41%, slightly higher than the value in 1985. SOCD value between 35 and 50 Mg C ha^−1^ accounted for 23.43%. 6.95% of soils had an SOCD value between 50 and 65 Mg C ha^−1^, this being 6.19% lower than that in 1985. Although only 12.84% of soils had an SOCD value less than 20 Mg C ha^−1^, this proportion was higher than that recorded in 1985. The proportion of soil with an SOCD value higher than 65 Mg C ha^−1^ was 5.37%, 0.76% lower than that in 1985; these soils were predominantly distributed in the eastern area of the province. Compared with the soil density distribution in 2015 and 1985, SOCD in northern Jilin Province significantly decreased. This decrease may be the reason that the area ratio of soils with higher organic carbon density decreased while that of soils with lower organic carbon density increased.

**Fig 7:**
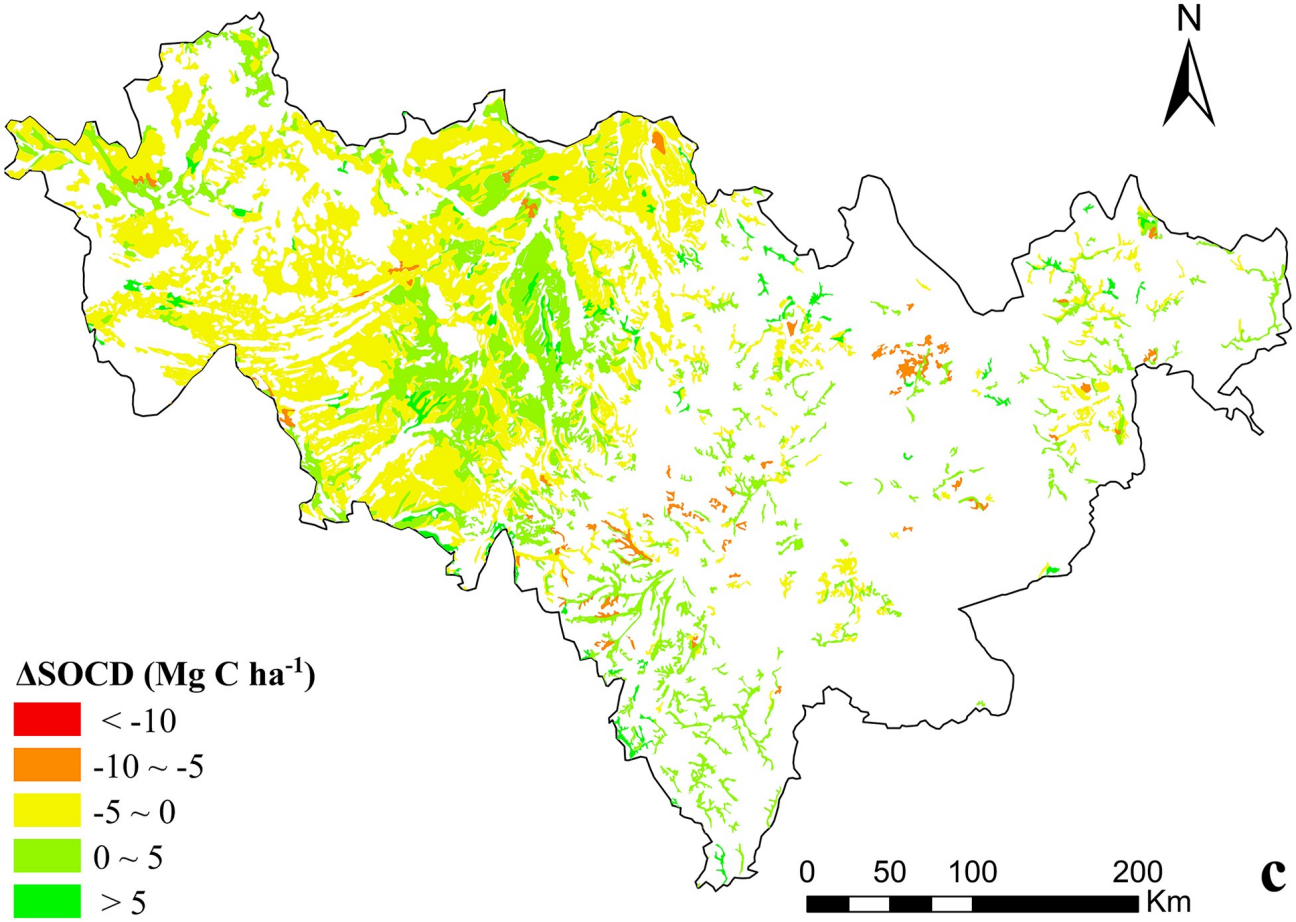
Spatial variability of ΔSOCD of dry farmland, Jilin Province, China.

Between 1985 and 2015, areas that recorded a decrease in SOCD accounted for 64.04% of Jilin province (Fig 7). In addition, areas which recorded an increase in SOCD ranging from -10 to -5 Mg C ha^−1^ and -5 to 0 Mg C ha^−1^ accounted for 2.58% and 60.13% of dry farmland, respectively. These areas were predominantly distributed in the central and western area of the province, respectively. Between 1985 and 2015, increases in SOCD accounted for 35.96%. The proportion of the increase of SOCD in 0–5 Mg C ha^−1^ was 32.56%. Soils with an SOCD increase greater than 5 Mg C ha^−1^ accounted for only 3.40% of the dry farmland.

Influenced by natural and economic conditions, obvious regional differences in agriculture were identified in the province. Agriculture and forestry are dominant in the eastern mountainous areas, and agriculture is dominant in central areas. In the western areas, agriculture and animal husbandry are dominant. Dry farmland in this province is mainly distributed in the central and western regions; distribution in the eastern region is relatively small. As SOCD in the surface soil of dry farmland has a high spatial variability, SOCD of the surface soil in dry farmland in Jilin Province gradually decreased gradually from the east to the west.

### SOC dynamics between soil groups

**Fig 8:**
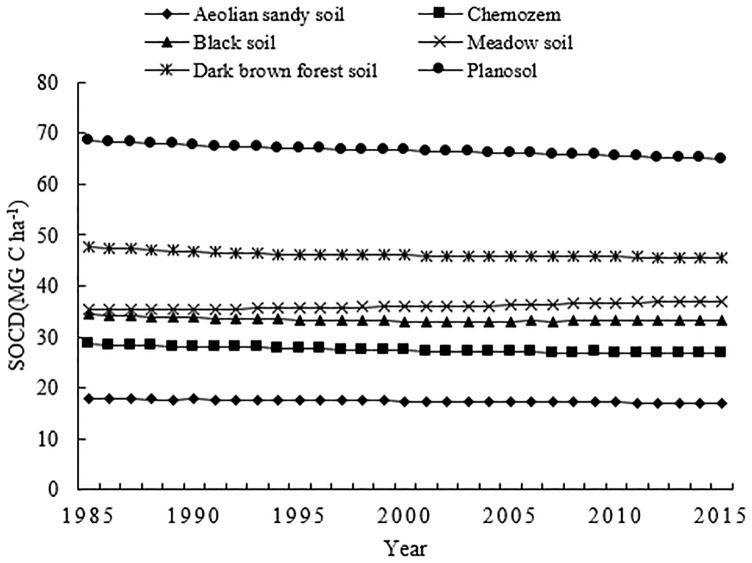
Changes of soil organic carbon density (SOCD) in the different soils of Jilin Province, China.

In this study, six soil types in Jilin Province were selected (Table 3), covering more than 90% of the area (Fig 8). In 1985, SOCD of each soil group were ranked as (high to low): planosol (68.48Mg C ha^−1^), dark brown forest soil (47.61Mg C ha^−1^), meadow soil (35.54Mg C ha^−1^), black soil (34.36Mg C ha^−1^), chernozem (28.60Mg C ha^−1^) and aeolian sandy soil (17.96Mg C ha^−1^).

**Table 3:** General information of different soil types.

Soil type	Sand	Silty sand	Clay particle	BD	SOM(g/kg)	pH
**Aeolian sandy soil**	78.36	9.72	11.92	1.46	9.50	8.10
**Black soil**	38.69	43.08	18.23	1.16	40.00	7.20
**Chernozem**	34.49	40.64	24.87	1.30	32.90	7.80
**Dark brown** **forest soil**	47.10	31.90	21.01	1.07	41.30	5.85
**Meadow soil**	33.39	46.55	20.07	1.30	33.55	5.70
**Planosol**	20.29	57.39	22.33	1.38	38.10	5.05

From 1985 to 2015, planosol, dark brown forest soil, black soil, chernozem and aeolian sandy soil recorded a decrease in SOCD value; only meadow soil recorded an SOCD increase (1.38 Mg C ha^−1^). Carbon lost in the five soil groups indicated a gradual temporal decrease in SOCD during this time period. The greatest average decrease was recorded by planosol (0.12 Mg C ha^−1^per year), the soil group which had the highest native SOCD. By comparison, dark brown forest soil, black soil, chernozem and aeolian sandy soil had average rates of decrease of 0.07, 0.04, 0.06, and 0.03 Mg C ha^−1^ per year, respectively. Chernozem, which is concentrated in Jilin Province, had the lowest native SOCD after aeolian sandy soil. Although the distribution area of planosol was small, its SOCD was higher than that of chernozem and black soil which had larger distribution areas.

Compared with 1985, an increase in carbon only occurred in meadow soil, total SOC increased by 1.95 Tg by 2015. Although the distribution area of meadow soil was limited (24.94%), it played a positive role in the carbon sequestration capacity of Jilin Province.

Reasons for these differences are not clear. Many studies have shown that soil organic matter content is affected by agricultural management measures, and has different sensitivity to different farmland management measures. This paper only describes the results and does not further analyze the reasons for the differences. It can be used as future research work.

### Uncertainty analyses

Models are commonly used to predict the dynamics of regional SOC. However, because input parameters in a model simulation unit are typically derived from a small amount of existing data, uncertainty is inevitable. In our study, accuracy in the model results was obtained by using soil data from the 1:1,000,000 soil database. However, in order to improve SOC dynamic results, other sources of uncertainty need to be considered.

It is impossible to obtain all the parameters needed for the model [31]. This lack of information can significantly affect the final model simulation result, and even reduce the accuracy of the simulation. Firstly, in the process of model simulation, the ratio of aboveground crops returning to soil in all polygons was the same (15%). If the ratio of crops to soil in each county is used, the accuracy of the simulation results will certainly be improved. Secondly, Land use data is important for modeling some SOC pools [13]. As it is difficult to obtain complete and reliable land use information before 1985, detailed original vegetation and historical agricultural production data are therefore difficult to obtain. To overcome this issue, a simplified method was adopted in the initialization of the model; this method however inevitably affected the simulation results. Thirdly, sensitivity analysis of the model [32, 33] indicates that the increment of SOC differed for different agricultural management measures. As sensitivity analysis was not undertaken in this study, it is important to undertake this in future studies.

In this study, as only data from one monitoring station was used to verify the model, this will inevitably affect the reliability of simulation results due to regional differences in soil type, climate etc. In order to improve the reliability of simulation results, regional verification is needed in future models.

## Conclusions

The results of point scale and regional verification indicated that the CENTURY model suitably simulated the dynamic change of SOC in Jilin Province.

Overall, approximately 35.96% of the total area played a positive role in carbon sequestration, with the area of carbon loss (64.04%) in the soil being larger than that of carbon sequestration. The content of SOCD in black soil area is similar to that of previous studies in Jilin Province. In both 1985 and 2015, areas with higher SOCD were concentrated in the eastern half of Jilin Province, and the change was more obvious in the northern part of Central Jilin Province. Soil types with a decrease of SOCD, such as meadow soil, black soil and chernozem, were mainly distributed in the west of Jilin Province, resulting in a significant decrease of SOCD in this area. The reason for the difference between the eastern and western regions may be related to the mode of agricultural development. In addition, SOC dynamics recorded apparently differences between different soil groups in Jilin. Soil groups with a higher initial SOCD lost carbon whilst all of the other soil groups recorded various levels of decrease over the past 30 years. In the future, it is necessary to further analyze the reasons for the differences.

Further exploration of farmland management measures to improve soil carbon sequestration capacity in Jilin Province is required for future research. In the future, it is necessary to analyze the reasons for the differences in order to improve the content of soil organic matter.

## Supporting information

S1 Table Modification of model parameters. (XLSX)

S1 File General information of soil profile. (XLS)

S1 Data (ZIP)

## References

[1] Yang YH, Su Y, He ZC, Yu M, Chen XJ, Shen AL. Transformation and distribution of straw-derived carbon in soil and the effects on soil organic carbon pool: A review. Chin. J. Appl. Ecol. 2019; 30:668–676. 10.13287/j.1001-9332.201902.026 30915820

[2] Sombroek WG, Nachtergaele FO, Hebel A. Amounts dynamics and sequestering of carbon in tropical and subtropical soils. AMBIO. 1993; 22:417–425. 10.1007/s13280-021-01508-y PMC8116452 33713291

[3] Wang Q, Song J, Cao L, Li XG, Yuan HM, Li N. Distribution and storage of soil organic carbon in a coastal wetland under the pressure of human activities. J. Soils Sediments. 2016; 17:1–12.

[4] Xu J, Su Y, Gao L, Cui XA. Review of the factors influencing soil organic carbon stability. Chin. J. Eco-Agric. 2018;26:222–230. 10.13930/j.cnki.cjea.170627

[5] Gao LP, Liang WJ, Jiang Y, Wen DZ. Dynamic change of organic carbon in black soil in northeast China using CENTURY model I. accumulation of soil organic carbon under natural conditions. Chin. J. Appl. Ecol. 2004; 15: 772–776. 15320391

[6] Foereid B, Høgh-Jensen H. Carbon sequestration potential of organic agriculture in northern Europe—a modelling approach. Nutr. Cycling Agroecosyst. 2004; 68:13–24.

[7] Lugato E, Berti A. Potential carbon sequestration in a cultivated soil under different climate change scenarios: a modelling approach for evaluating promising management practices in northeast Italy. Agri. Eco. Environ. 2008; 128:97–103.

[8] Galdos MV, Cerri CC, Cerri CEP, Paustian K, Van AR. Simulation of soil carbon dynamics under sugarcane with The CENTURY model. Soil Sci. Soc. Am. 2009; 73:802–811. 10.2136/sssaj2007.0285

[9] Tornquist CG, Mielniczuk J, Cerri CEP. Modeling soil organic carbon dynamics in oxisols of Ibiruba´ (Brazil) with the CENTURY model. Soil Till. Res. 2009b; 105:33–43 (2009b).

[10] Sommer R, Bossio D. Dynamics and climate change mitigation potential of soil organic carbon sequestration. J. Environ. Manage. 2014; 144:261–272. 10.1016/j.jenvman.2014.05.017 24929498

[11] Smith WN, Desjardins RL, Pattey E. The net flux of carbon from agricultural soils in canada 1970–2010. Glob. Change Biol. 2000; 6:557–568. 10.1046/j.1365-2486.2000.00340.x

[12] Falloon P, Smith P, Szab´o J, P´asztor L. Comparison of approaches for estimating carbon sequestration at the regional scale. Soil Use Manage. 2002; 18:164–174. 10.1111/j.1475-2743.2002.tb00236.x

[13] Ardö J, Olsson L. Assessment of soil organic carbon in semiarid Sudan using GIS and the CENTURY model. Arid Environ. 2003; 54:633–651.

[14] Lufafa A, Bolte J, Wright D, Khouma M, Diedhiou I, Dick RP, et al Regional carbon stocks and dynamics in native Woody Shrub Communities of senegal’s peanut basin. Agri. Eco. Environ. 2008; 128:1–11. 10.1016/j.agee.2008.04.013

[15] Tornquist CG, Gassman PW, Mielniczuk J, Giasson E, Campbell T. Spatially explicit simulations of soil C dynamics in southern Brazil: integrating CENTURY and GIS with i-Century. Geoderma. 2009; 150:404–414.

[16] Chen C, Wang J, Pan XB, Wei YR, Feng LP. Validation and adaptability evaluation of grass ecosystem model CENTURY in Inner Mongolia. Acta Agrestia Sin. 2012; 20:1011–1019.

[17] Yu FY. Simulation of soil organic carbon dynamics in desert steppe based on CENTURY model. D. Inner Mongolia Agricultural University, Hohhot, Inner Mongolia, 2018.

[18] Sun T, Ma QL, Li YK, Zhang YH, Wang YL, Guo CX. Simulation and analysis on the dynamics of soil organic carbon of wolfberry forest in secondary Saline-alkali land based on the CENTURY model. J. Anhui Agric. Sci. 2015; 43:202–206.

[19] Wang XY, Li YQ, Lian J, Luo YQ, Niu YY, Gong XW. Progress in application of the CENTURY model for prediction of soil carbon levels in different ecosystems. Acta Pratac. Sin. 2019; 28:179–189. 10.11686/cyxb2018126

[20] Xu WQ, Chen X, Luo GP, Zhang Q, Zhang YF, Tang F. The impact of land reclamation and management practices on the dynamics of soil organic carbon in the Arid region of North-western China as simulated by CENTURY model. Acta Ecol. Sin. 2010; 30:3707–3716.

[21] Xie Z, Zhu J, Liu G. Soil organic carbon stocks in China and changes from 1980s to 2000s. Glob. Change Biol. 2007; 13:1989–2007. 10.1111/j.1365-2486.2007.01409.x

[22] Wang DY. Simulation and prediction of soil organic carbon spatial change in Arable Lands Based on DNDC model. D. Chinese Academy of Agricultural Sciences, Beijing, 2014.

[23] Yu SS, Dou S, Huang J, Yang JM, Shi Y, Zheng HY. Organic carbon storage of cultivated top soils and its influencing factors in Jilin Province. J. Agro-Environ. Sci. 2014; 33:1973–1980.

[24] Shi XZ, Yu DS, Warner ED, Pan XZ, Petersen GW, Gong ZG, et al Soil database of 1:1 000 000 digital soil survey and reference system of the Chinese Genetic Soil Classification System. Soil Sur. Horizons. 2004; 45:129–136.

[25] Food and Agriculture Organization of the United Nations (FAO). 1988. FAO/UNESCO Soil Map of the World. Revised Legend. World Soil Resources Report 60. FAO, Rome.

[26] Parton WJ, Stewart JWB, Cole CV. Dynamics of C, N, P and S in grassland soils: A Model. Biogeochemistry. 1988; 5:109–131.

[27] Zhao YC, Shi XZ, Weindorf DC, Yu DS, Sun WX, Wang HJ. Map scale effects on soil organic carbon stock estimation in North China. Soil Sci. Soc. Am. 2006; 70: 1377–1386.

[28] Ogle SM, Breidt FJ, Easter M, Williams S, Killian K, Paustian K. Scale and uncertainty in modeled soil organic carbon stock changes for US croplands using a process-based model. Glob. Change Biol. 2010; 16:810–822. 10.1111/j.1365-2486.2009.01951.x

[29] Tang J, Xu XM, Li ZY, Zhao N, Luo R, Han WZ. Soil organic carbon storages and their spatial and temporal distribution in Tongyu County, Jilin Province. Quaternary Sciences. 2010; (3):584–590.

[30] Liao Y, Sun SM, Yang ZF, Xia XQ, Bai RJ. Soil organic carbon storage and its spatial-temporal variation in the central and western area of Jilin. Quaternary Sciences. 2011; (1):189–198.

[31] Wang SH, Wang XZ, Shi YC, Zhao DC, Weindorf DS, Yu SX, et al Regional simulation of soil organic carbon dynamics for dry farmland in East China by coupling a 1:500 000 soil database with the Century Model. Pedosphere. 2011; 21(3):277–287.

[32] Wang XB, Cai DX, Hoogmoed WB. Scenario analysis of tillage, residue and fertilization management effects on soil organic carbon dynamics. Pedosphere. 2005; 15:473–483.

[33] Paustian K, Parton WJ, Person J. Modeling soil organic matter in organic-amended and nitrogen-fertilized long-term plots. Soil Sci. Soc. Am. J. 1992; 56:476–488.

